# Lack of association of *DRD3* and *CNR1* polymorphisms with premenstrual dysphoric disorders

**Published:** 2015-04

**Authors:** Mesut Yıldız, Mehmet Vural, Mehmet Emin Erdal, Özlem İzci Ay, Şenay Görücü Yılmaz, İbrahim Fatih Karababa, Salih Selek

**Affiliations:** 1 *Department of Psychiatry, Faculty of Medicine, Gaziosmanpaşa University, Tokat, Turkey.*; 2 *Department of Obstetrics and Gynecology, Faculty of Medicine, Harran University, Sanliurfa, Turkey.*; 3 *Department of Medical Biology and Genetics, Faculty of Medicine, Mersin University, Mersin, Turkey.*; 4 *Department of Psychiatry, Faculty of Medicine, Harran University, Sanliurfa, Turkey.*; 5 *Department of Psychiatry &Behavıoral Sciences, University of Texas Health Science Center at Houston, Houston, Texas, USA.*

**Keywords:** *Cannabinoid receptor*, *Dopamine D3 receptor*, *Premenstrual syndrome*, *Genetic polymorphism*

## Abstract

**Background:**

Premenstrual dysphoric disorder (PMDD) is a mood disorder characterized with physical and affective symptoms during the luteal phase of susceptible women.

**Objective:**

The aim of this study was to investigate the association of Dopamine D3 receptor (*DRD3*) polymorphism, and Cannabinoid receptor Type 1 (*CNR1*) polymorphism with PMDD.

**Materials and Methods:**

Fifty one participants with documented PMDD according to the DSM IV criteria and 51 healthy controls were included in this cross sectional study. Symptom severity was measured with daily self-rating, monthly premenstrual assessment forms and psychiatric interviews. The genotyping of DRD3 receptor and Cannabinoid type 1 receptors were performed using Taqmanfluorogenic assay method.

**Results:**

Distribution of *DRD3* and *CNR1* polymorphism was not different between patients and controls.

**Conclusion:**

These findings do not support a major role of *DRD3*, and *CNR1* polymorphisms in contributing to susceptibility to premenstrual dysphoric disorder.

## Introduction

Premenstrual syndrome (PMS) is characterized by recurrent psychological and/or somatic symptoms occurring specifically during the luteal phase of the menstrual cycle and resolving during menstruation. Premenstrual dysphoric disorder (PMDD) is the extreme, predominantly psychological end of the PMS spectrum and it is estimated that 5-10% of regularly ovulating women experience PMDD (1). Key features include depressed mood, anxiety, affective liability, persistent anger or irritability, and change in appetite or sleep (2). The cause of PMDD is unknown. Studies attempting to elucidate the pathophysiology of the syndrome concentrate on the hypothalamic- pituitary- adrenal (HPA) axis, the γ-amino butyric acid (GABA) system, the serotonergic system, and the opioid system (3).

Several lines of evidence suggest that deregulation of serotonergic transmission is involved in the pathophysiology of PMDD (4-6). Inhibition of serotonergic activity has been shown to aggravate symptoms of premenstrual dysphoric disorder. Furthermore, metergoline, a serotonin- selective antagonist that blocks serotonin (5-HT) receptors (particularly 5-HT2A and 5-HT2C) also provokes a return of symptoms in women with premenstrual dysphoric disorder treated with fluoxetine (7). PMDD is included under the category of "depressive disorders not otherwise specified" in DSM-IV. PMDD has a high comorbidity with **other axis I disorders **such as major depressive disorder, bipolar disorder, and anxiety disorders (8-10). Premenstrual symptoms were reported by twice as many women diagnosed with mood disorders (68%) than women without a psychiatric diagnosis (34%) (10). It has been shown that, 38-46% of women with PMDD have comorbid seasonal affective disorder and 11-38% report a comorbid anxiety disorder (8).

There is an overlap in the symptoms experienced by women with PMDD and patients with other mood disorders. As in patients with other mood disorders, past episodes of mood disorder and family history of mood disorder are common in women with PMDD (11). Family-linkage and twin studies have indicated that genetic factors often play an important role in the development of mental disorders. Evidence from family and twin studies suggests that there is a significant genetic contribution to premenstrual dysphoric disorder (12-14). Recent studies have been intensified for possible association between PMDD and candidate genes of the serotonergic system. Studies to date have searched for polymorphism in the serotonin transporter (*SLC6A4*) including the 5’HTTLPR, serotonin transporter promoter rs25531, serotonin receptor 1A C (-1019) G, and activating protein 2β (AP-2β) (18) and found no positive results (15-18).

The dopamine D 3 receptor gene (*DRD3*) is candidate for a number of psychiatric conditions including schizophrenia, bipolar disorder, and alcohol and drug abuse (19). The (*DRD3*) gene affects patients with major depressive disorder and their response to antidepressant treatment (20, 21). *Rs6280*, also known as Ser9Gly, is a SNP in the *DRD3 *gene. The *Rs6280 *(C) allele encodes glycine, and the (T) allele encodes serine. Studies to date have shown that polymorphisms in *DRD3* gene have associations with schizophrenia, depression, nicotine dependence, and attention deficit-hyperactivity disorder (22-25). 

A preliminary study showed that *DRD3*Ser9Gly polymorphism affected response to antidepressant treatment in major depressive disorder (23). The endocannabinoid system is widely distributed throughout the brain and modulates many functions. The cannabinoid receptors are a class of cell membrane receptors under the G protein-coupled receptor superfamily (26). There are currently two known subtypes, termed CB1 and CB2 (27). The CB1 (CNR1) receptor is expressed mainly in the brain. The endocannabinoid receptor type 1 gene, *CNR1* located on 6q14-q15. The endocannabinoid system is involved in mood and related disorders (28). Activation of CB1 receptors can be used for the treatment of pain, anxiety, depression and post-traumatic stress disorder (29). Genetic polymorphisms of the endocannabinoid system have been explored in mental disorders. *CNR1* polymorphisms were found to be associated with substance use disorders, depression, anxiety disorders, eating disorders, schizophrenia, and attention deficit hyperactivity disorder (30-37). Many single nucleotide polymorphisms (SNPs) have been identified at the *CNR1* locus so far (38). 

rs1049353 and rs12720071 are common variants of *CNR1* gene. Based on a study of 256 Caucasian patients being treated for depression, carriers of a rs1049353 (G) allele were less likely to respond favorably, particularly if they were females with comorbid anxiety (39). There are three genotypes (GG, GA, AA) for *CNR1* gene (rs1049353). Since polymorphisms in *DRD3Ser9Gly* and *CNR1 *receptors are seemed to be associated with anxiety and depressive disorders and as it is known that PMDD shares a range of characteristics with depressive and anxiety disorders; our aim was to investigate whether *DRD3Ser9Gly* and *CNR1* receptor polymorphisms are related to PMDD or not.

## Materials and methods

**Study population**

The cross-sectional study was approved by the Harran University Local Ethics Committee, and informed written consents were obtained from each participant. Patients were recruited from consecutive application to the Harran University Research Hospital, Obstetrics and Gynecology Outpatient Clinic, Sanliurfa, Turkey in 2011. The control group was selected from the staff of Faculty of Medicine. 51 patients with PMDD and 51 healthy control subjects between the ages of 18 and 45 years were included to the study. 

All participants reported regular menstrual cycles and none was taking oral contraceptives, hormone replacement therapy or psychotropic drugs. Any women known to have an existing Axis I psychiatric disorder according to the DSM IV criteria was excluded from the study. Clinical diagnosis was determined by precise diagnostic criteria that were outlined in the 4^th^ Edof the Diagnostic and Statistical Manuel of Mental Disorders (DSM-IV). DSM-IV criteria for PMDD require the presence of 5 of 11 specific diagnostic symptoms.

These symptoms should be limited to the luteal phase and should not represent amplification of preexisting depression, anxiety, or personality disorder. In addition, they must be confirmed prospectively by daily rating for at least two consecutive menstrual cycles. Control subjects reported no significant premenstrual symptoms. All subjects were evaluated with a semi-structured interview form; which was used to determine the sociodemographic features of the participants. This form also evaluates the symptoms of PMS, family history of PMS and nicotine use. Clinical categorization of PMDD patients and control subjects was determined by prospective symptom rating with the use of the Daily Record of Severity of Problems (DRSP) scale-short form, based on self-assessment reports spanning two consecutive menstrual cycles (40). The DRSP scale consists of eleven questions evaluating the DSM-IV diagnostic criteria for PMDD and 3 questions asking for the effects of these symptoms on functionality. This study is a cross-sectional study investigating the association between *DRD3* and CNR1 receptor polymorphisms and PMDD.

**Procedures**

Venous blood samples were collected in ethylenediaminetetraacedic acid (EDTA) containing tubes. DNA was extracted from peripheral blood leukocytes by salting out procedure (41).

Genotypic analysis of *DRD3* Gene Ser9Gly (rs6280) polymorphism:

Genotypes were determined using a TaqMan™ fluorogenic 5′-nuclease assay with TaqMan Probes. All reactions were carried out following the manufacturer’s protocol. Primer Express 3.0 (Applied Biosystems) was used to design both the PCR primers and the TaqMan probes. For the *DRD3* gene Ser9Gly, *rs6280* polymorphism custom made primers and probes are as follows: Forward primer 5’-TCCCTCTGGGCTATGGCAT-3’, Reverse primer 5’- GCTGGCACCTGTGGAGTTCT-3’, ProbeG(C)5’- YakimaYellow- TGAGTGG(pdC) CA (pdC) CTGAA (pdC) TACA-BHQ-1-3’ and Probe A(T) 5’-FAM- AG(pdC)TGAGTAG(pdC) CA(pdC)CTGAA(pdC)TA-BHQ-1-3’ (Metabion International AG, D-82152 Martinsried/ Deutschland). Single nucleotide polymorphism amplification assays were performed according to the manufacturer’s instructions. In brief, 25µl of reaction solution containing 30 ng of DNA was mixed with 12.5µl of 2X TaqMan Universal PCR Master Mix (Applied Biosystems) , 900 nmol of each primer, and 200 nmol of each probe. Reaction conditions consisted of preincubation at 60ºC for 1 min and at 95ºC for 10 min, followed by 40 cycles at 95ºC for 15 sec and at 60ºC for 1 min. Amplifications and analysis were performed in an ABI Prism 7500 Real-Time PCR System (Applied Biosystems), using the SDS 2.0.3 software for allelic discrimination (Applied Biosystems).

Genotypic analysis of *CNR1* 1359 G>A (codon Thr453Thr, rs1049353) polymorphisms:

The genotyping of *CNR1* 1359 G>A (codon Thr453Thr, rs1049353) polymorphisms was performed using predesigned TaqMan SNP Genotyping Assays (Applied Biosystems, Foster City, CA). The Assays-on-Demand SNP genotyping kit was used for the polymerase chain reaction (Applied Biosystems Real Time PCR Systems Foster City, California). Single nucleotide polymorphism amplification assays were performed according to the manufacturer’s instructions.

In brief, 25µl of reaction solution containing 30 ng of DNA was mixed with 12.5µl of 2X TaqMan Universal PCR Master Mix (Applied Biosystems) and 1.25 µl of predeveloped assay reagent from the SNP genotyping product (C_1652590_10 for *CNR1* 1359 G>A, codon Thr453Thr, rs1049353, Applied Biosystems) containing two primers and two MGB TaqMan probes. Reaction conditions consisted of preincubation at 60ºC for 1 min and at 95ºC for 10 min, followed by 40 cycles at 95ºC for 15 sec and at 60ºC for 1 min. Amplifications and analysis were performed in an ABI Prism 7500 Real-Time PCR System (Applied Biosystems), using the SDS 2.0.3 software for allelic discrimination (Applied Biosystems). All procedures were conducted in a manner blind to the case status and other characteristics of the participants. Scoring of gels and data entry was conducted independently by two persons.

**Statistical analysis**

All statistical analysis was performed using the Statistical Package for Social Sciences for windows 11.0 (SPSS, Chicago, IL). The ^2^ tests were performed to assess conformity to Hardy-Weinberg equilibrium and to detect any association between each genotype distribution and clinical category. Statistical significance was considered at exact probability values of p<0.05.

## Results

In total 51 patients with PMDD (age range: 20-46 years; mean= 30.2) and 51 healthy control subjects (age range: 15-44 years, mean= 28.0) were included in the study. There was no significant difference in age, BMI, height, weight, and number of children between PMDD group and controls except for marriage rates. 5.9% of PMDD patients were single, while 29.4% of the controls were single. Table I shows the demographic data of the patients and control group. Allele and genotype frequencies were not different between PMDD patients and controls in *DRD3Ser9Gly* polymorphism ( ^2^=0.356, and p=0.837). Table II shows the genotype distribution of *DRD3Ser9Gly* in the PMDD and control groups.

Allele and genotype frequencies were not different between PMDD patients and controls in CNR1 polymorphism. Table III shows the genotype distribution of *CNR1* polymorphism in the PMDD and control groups. Genotypes have Hardy-Weinberg equilibrium in *DRD3Ser9Gly* in the PMDD group (^2^=1.65 with 1 DF) but the other genotypes are not in Hardy-Weinberg equilibrium.. There was not a significant difference of *DRD3Ser9Gly* polymorphism between PMDD patients and controls. There was not a significant difference of *CNR1* polymorphism between PMDD patients and controls.

**Table I:** Demographic data of the PMDD patients and control subjects

	**PMDD patients** **(n=51)**	**Controls** **(n=51)**
Age (Year)	30.27 ± 5.84	28.07 ± 7.42
BMI (Kg/m²)	25.83 ± 3.57	25.34 ± 4.61
Height (cm)	162.23 ± 5.25	163.07 ± 6.92
Weight (Kg)	68.29 ± 11.72	67.19 ± 12.08
NoC	1.35 ± 1.62	1.13 ± 1.70
MaR )%(	94.11	70.58

Results are expressed as mean±SD

N: Number

BMI: Body Mass Index

NoC: Number of Children

MaR: Marriage rate

**Table II:** Genotype frequencies of the *DRD3*polymorphism in PMDD and healthy control subjects

**Polymorphism**	**PMDD patients** **(n=51)**	**Controls** **(n=51)**
T/T (Ser/Ser)	26	23
T/C (Ser/Gly)	18	20
C/C (Gly/Gly)	7	8

The data was analyzed with Chi-square’s test. (p=0.085)

Allele and genotype frequencies were not different between PMDD patients and controls in *DRD3* polymorphism.

Ser: Serine

Gly: Glycine

**Table III:** Genotype frequencies of the *CNR1* polymorphism in PMDD and healthy control subjects

**CNR1 polymorphism**	**PMDD patients** **(n=51)**	**Controls** **(n=51)**
C/C (Gly/Gly)	45	36
C/T (Gly/Ser)	4	11
T/T (Ser/Ser)	2	4

The data was analyzed with Chi-square test (p=0.837)

Allele and genotype frequencies were not different between PMDD patients and controls in CNR1 polymorphism.

Ser: Serine

Gly: Glycine.

## Discussion

We genotyped the *DRD3Ser9Gly* (rs6280) and *CNR1* polymorphisms in two groups of regularly ovulating women, one group with clinically diagnosed premenstrual dysphoric disorder and one group of normal healthy controls with no symptoms of premenstrual dysphoria. We found no association of *DRD3Ser9Gly* (rs6280) polymorphisms in PMDD. The D3 receptor is candidate for being involved in mental disorders. Polymorphisms in the *DRD3* gene have been studied in various psychiatric disorders. In a study of 88 patients being treated for schizophrenia with olanzapine, those who were rs6280 (C; C) homozygotes had greater positive symptom remission as compared with (C; T) or (T; T) genotypes (42). The Ser9Gly polymorphism has been associated with depression in different studies (43-45).

A preliminary study showed that *DRD3Ser9Gly* polymorphism affected response to antidepressant treatment in major depressive disorder (28). Pharmacogenetic studies have reported that *DRD3Ser9Gly* polymorphism influenced antidepressant response in bipolar disorder patients treated with a combination of olanzapine and fluoxetine (44). Our first finding is lack of an association of *DRD3Ser9Gly* polymorphism in PMDD and there is no other study looking for this association. As the etiology of PMDD is multifactorial, dopaminergic pathways may not be sole responsible in the pathophysiology of PMDD. Our second finding is lack of association between *CNR1* polymorphism and PMDD. The endocannabinoid system has been implicated in the pathogenesis of depression and anxiety. Patients with depression are found to have reduced levels of circulating endocannabinoids and an up-regulation of CN1R was observed in the prefrontal cortex of subjects with major depression who died by suicide (46). Since *CNR1* polymorphism (rs1049353) is associated with depression and anxiety, we did not find an association between *CNR1* polymorphism (rs1049353) and PMDD. Endocannabinoid system may not be the sole responsible in the pathophysiology of PMDD. The previous genetic studies in premenstrual dysphoric disorder were mostly about the serotonergic and noradrenergic systems. To our knowledge, this study is the first reported genotypic analysis of *DRD3Ser9Gly* (rs6280) and *CNR1* polymorphisms in premenstrual dysphoric disorder. There may be several explanations for our negative findings.

First, clinical categorization of patients with PMDD can be difficult because of the subjective nature of symptom interpretation. Second limitation is the possibility of population stratification. In studies comprising subjects taken primarily from a localized community, it is important to include healthy controls to determine typical genotype and allelic frequencies, although these may not be representative of the wider population. Third, the lack of association between the *DRD3Ser9Gly* (rs6280) and *CNR1 *polymorphisms and PMDD may be affected by sample size. We were unable to identify either a single genetic marker or a combined polymorphic profile for susceptibility to PMDD. 

However, it is the first study evaluating *DRD3Ser9Gly* and *CNR1* polymorphisms in PMDD. It is not feasible to expect a single polymorphism to be the sole factor that is responsible for PMDD. It is likely that PMDD is a polygenic disorder, but the relative contributions of the various implicated genes are unknown. Cautious interpretation of the present study is warranted, both by the preliminary nature of these findings and by their basis in simple association analysis. Within the limits that are imposed by the sample size, the polymorphisms that were studied here do not represent major risk factors for PMDD. Confirmation of our findings will require independent validation in a larger group of subjects.

## Conflict of interest

The authors declare that there is no conflict of interests regarding the publication of this paper.
